# Elevated Preattentive Affective Processing in Individuals with Borderline Personality Disorder: A Preliminary fMRI Study

**DOI:** 10.3389/fpsyg.2015.01866

**Published:** 2015-12-02

**Authors:** Arielle R. Baskin-Sommers, Jill M. Hooley, Mary K. Dahlgren, Atilla Gönenc, Deborah A. Yurgelun-Todd, Staci A. Gruber

**Affiliations:** ^1^Mechanisms of Disinhibition, Department of Psychology, Yale University, New HavenCT, USA; ^2^Department of Psychology, Harvard University, CambridgeMA, USA; ^3^Brain Imaging Center, McLean Hospital, BelmontMA, USA; ^4^Department of Psychiatry, Harvard Medical School, BostonMA, USA; ^5^Cognitive Neuroimaging Laboratory, The Brain Institute, University of Utah, Salt Lake CityUT, USA

**Keywords:** borderline personality disorder, emotion, backward masked affect, preattentive

## Abstract

**Background:** Emotion dysregulation is central to the clinical conceptualization of borderline personality disorder (BPD), with individuals often displaying instability in mood and intense feelings of negative affect. Although existing data suggest important neural and behavioral differences in the emotion processing of individuals with BPD, studies thus far have only explored reactions to *overt* emotional information. Therefore, it is unclear if BPD-related emotional hypersensitivity extends to stimuli presented *below* the level of conscious awareness (preattentively).

**Methods:** Functional magnetic resonance imaging (fMRI) was used to measure neural responses to happy, angry, fearful, and neutral faces presented preattentively, using a backward masked affect paradigm. Given their tendency toward emotional hyperreactivity and altered amygdala and frontal activation, we hypothesized that individuals with BPD would demonstrate a distinct pattern of fMRI responses relative to those without BPD during the viewing of masked affective versus neutral faces in specific regions of interests (ROIs).

**Results:** Results indicated that individuals with BPD demonstrated increases in frontal, cingulate, and amygdalar activation represented by number of voxels activated and demonstrated a different pattern of activity within the ROIs relative to those without BPD while viewing masked affective versus neutral faces.

**Conclusion:** These findings suggest that in addition to the previously documented heightened responses to overt displays of emotion, individuals with BPD also demonstrate differential responses to positive and negative emotions, early in the processing stream, even before conscious awareness.

## Introduction

Borderline personality disorder (BPD) is characterized by severe disruptions in self-image, problems with emotion and behavior regulation, and difficulties in the maintenance of functional interpersonal relationships (American Psychiatric Association [APA], 2013). Comprising up to 20% of psychiatric inpatient populations and 2% of the general population (Skodol et al., 2002), individuals meeting diagnostic criteria for BPD represent a significant challenge for clinicians and researchers focused on psychopathology. One of the central features of BPD that interferes with adaptive functioning and treatment progress is emotion dysregulation.

Both clinical conceptualizations and research studies indicate that individuals with BPD have heightened sensitivity to emotion stimuli, greater intensity of emotion experience, a slow return to emotional baseline, and engage in their most acute problematic behaviors when in emotional contexts (Linehan, 1993; Chapman et al., 2008; Chapman et al., 2010; Sprague and Verona, 2010). Abnormalities in the processing of emotionally relevant information have been identified in individuals with BPD using self-report measures (Levine et al., 1997; Henry et al., 2001; Koenigsberg et al., 2009), behavioral tasks (Wagner and Linehan, 1999; Hochhausen et al., 2002; Bland et al., 2004; Dyck et al., 2009), and functional neuroimaging (Herpertz et al., 2001; Donegan et al., 2003; Johnson et al., 2003; Hooley et al., 2010). Overall, individuals with BPD report greater mood lability and emotion intensity than individuals with other personality disorders or Bipolar II disorder (Henry et al., 2001). Interestingly, however, some studies suggest that the heightened sensitivity reported by individuals with BPD may be exclusive to negative emotions (Bland et al., 2004; Domes et al., 2009; Ruocco et al., 2013). Specifically, this research found that individuals with BPD display higher levels of reactivity to negative affect, anger, and anxiety, but similar levels of positive affect reactivity compared to individuals without BPD and other personality disorders.

Consistent with these findings, evidence suggests that individuals with BPD display preferential sensitivity to subtypes of negatively valenced emotion on behavioral tasks, such as negative words (Arntz et al., 2000), stimuli displaying borderline-related words (e.g., abandon et al., suicidal, alone; Korfine and Hooley, 2000), and negative emotions (Levine et al., 1997; Wagner and Linehan, 1999; Bland et al., 2004). For example, a study by Domes et al. (2008) presented faces that were ambiguous blends of different facial expressions to individuals with and without BPD. Individuals with BPD significantly over-reported the presence of anger in these faces. Veague and Hooley (2014) also have reported evidence of the misidentification of anger in faces containing no anger cues among individuals diagnosed with BPD. Additionally, consistent evidence exists that suggests patients with BPD misattribute negative emotions to neutral faces (Daros et al., 2013, 2014). Together, these studies suggest that individuals with BPD display heightened responsivity toward facial emotion expressions and a tendency to visually perceive negative emotion, even when it is not explicitly represented.

Beyond their preferential sensitivity, some research suggests that individuals with BPD also tend to be less accurate in identifying overt expressions of negative emotion (e.g., anger, disgust, sadness, and fear) while viewing pictures of faces (Levine et al., 1997; Bland et al., 2004). Moreover, Bland et al. (2004) reported that accuracy in identifying emotions was inversely correlated with self-reported negative affect in daily life. While substantial behavioral evidence indicates that those with BPD display deficits in emotion recognition, there is some debate as to whether these deficits are consistent with a model of emotion hypersensitivity or impairment in labeling emotions accurately and a bias toward negative emotions (Domes et al., 2009).

Regardless of the specific mechanism influencing the BPD-related heightened emotion reactivity, in general, neuroimaging studies provide support for the presence of a heightened responsivity to emotional stimuli among individuals with BPD. Given the crucial role of frontal and limbic areas in emotion processing and regulation (LeDoux, 2000), researchers examining the neurophysiology of emotional dysfunction in BPD have tended to focus on the amygdala and frontal cortex (Goldin et al., 2008; Etkin et al., 2011). More specifically, abnormalities in these regions have been reported within BPD individuals and are likely to play a role in the severe disruptions in emotional functioning and failures of inhibitory control observed in BPD (Korfine and Hooley, 2000; Ruocco et al., 2013). An initial study found increased blood oxygen level-dependent (BOLD) responses in the bilateral amygdala in response to negative stimuli from the International Affective Picture System (IAPS) in BPD (Herpertz et al., 2001). Enhanced neural activation of the right amygdala in BPD in response to fearful faces compared to neutral faces has also been reported (Minzenberg et al., 2007). More recently, Hazlett et al. (2012) reported that individuals with BPD displayed enhanced amygdala activity to emotional (unpleasant and pleasant) but not neutral pictures and a prolonged return to a hemodynamic baseline after viewing the pictures. Whereas a number of imaging studies report increased amygdala activation specifically to negatively valenced information, one study found that left-sided amygdala hypersensitivity occurred regardless of the specific stimulus valence in a sample of patients with BPD (Donegan et al., 2003) and a recent meta-analysis reported reduced right amygdala activation when comparing negative to neutral images in BPD patients, with only the potential for post-traumatic stress disorder as a diagnostic comorbidity, versus controls (Ruocco et al., 2013).

In addition to the alterations noted within the amygdala, BPD-related differences in frontal activation are consistently reported. For example, a handful of studies report greater frontal activation, particularly within the lateral frontal cortex in response to images across valence or to pleasant images, specifically (Herpertz et al., 2001; Koenigsberg et al., 2009). However, in response to negative pictures, there is evidence of attenuation in the lateral frontal cortex (Ruocco et al., 2013) and rostral anterior cingulate cortex (Minzenberg et al., 2007). In general, despite some inconsistency in results with regard to amygdalar and frontal activation, it appears that, overall, individuals with BPD demonstrate differences in the perception and processing of emotionally laden stimuli. Thus far, neuroimaging studies largely have provided information consistent with self-report and behavioral studies and relevant to an understanding of emotional dysfunction in BPD. However, neuroimaging research in this area is still in its early stages.

As reviewed above, only a few studies on BPD have utilized neuroimaging methods and all thus far have examined emotion reactivity using overt affective information. Substantial evidence exists, however, indicating that emotion is first appraised and evaluated at a preconscious, automatic level. If individuals with BPD have a propensity toward emotional hypersensitivity, it is possible that this heightened sensitivity to perceive and neurally detect affective information might be evident even prior to the explicit representation of emotion information. The primary aim of this study was to explore the neural response of individuals with and without BPD, while completing a paradigm that presented emotional faces below the level of conscious awareness (i.e., masked facial affect task; e.g., see Balconi and Mazza, 2009; Gruber et al., 2009; Viding et al., 2012; McCrory et al., 2013; Sagar et al., 2013 for examples in other populations). Preattentive processing is performed automatically in visual regions, though is reflected in neural areas commonly involved in affective processing, including the amygdala and frontal regions (Ohman, 2008). Given the increased emotion reactivity to negatively and positively valenced information often noted in individuals with BPD and previous findings of altered amygdala and frontal activation in this group, we hypothesized that individuals with BPD would demonstrate a distinct pattern of neural activation relative to individuals without BPD in response to affective stimuli, even when presented below the level of conscious processing.

## Materials and Methods

### Participants

Thirteen female adults diagnosed with BPD and eleven female adults without a diagnosis of BPD, who were age (mean = 25.21, *SD* = 4.48) and education matched (all participants were college educated or currently in college), were included in the study. Participants were recruited from the greater Boston area by means of advertisements in local media. All participants were part of a larger study and completed multiple measures and experimental tasks during their study visit (Hooley et al., 2010). All participants received a Structured Clinical Interview for DSM Disorders (First et al., 1997a,b) assessment conducted by a trained rater for Axis I and II disorders. To be included as a healthy control, participants had to be free of current or past Axis I or Axis II pathology and also had to report no symptoms of BPD (i.e., none of the 11 healthy controls met clinical threshold for any of the SCID-II BPD items). All BPD participants met DSM-IV (and now also DSM-5) diagnostic criteria for the disorder (i.e., at least 5 of 9 symptoms; Mean = 6.77 symptoms, *SD* = 1.30). Participants were excluded if they reported a history of head trauma or neurological problems. Medication use or other current Axis I or Axis II disorders were not used as exclusion criteria for BPD participants. Five of the 13 BPD participants were diagnosed with current major depression and 12 of 13 reported a past history of major depression. No participant with BPD had a current or past history of PTSD. Other clinical problems, such as eating disorders, social phobia, dysthymia, and past drug/alcohol abuse, were common in this sample (see Hooley et al., 2010 for additional clinical information on this sample). Ten of the BPD participants were taking antidepressant medications. Prior to their participation in any study related activity, study procedures were explained and all participants read and signed an informed consent form. This described in detail all study and scanning procedures, which had been approved by the McLean Hospital Institutional Review Board.

### Masked Facial Affect Task

Backward masked affect refers to a phenomenon wherein presenting one visual stimulus (a “mask”) immediately after another brief “target” visual stimulus leads to a failure to consciously perceive the first stimulus. The masked facial affect task stimuli were comprised of faces obtained from the picture set from the Neuropsychiatry Section of the University of Pennsylvania (Erwin et al., 1992) and consisted of black and white photographs of males and females posing each of three different emotional states (happy, anger, fear) and posing with neutral facial affect. Both emotional and neutral faces were used as the target stimuli and neutral faces were used as masking stimuli. All masking stimuli were matched to the target stimuli, meaning that they were images of the same individual presented during the target stimuli.

**FIGURE 1:**
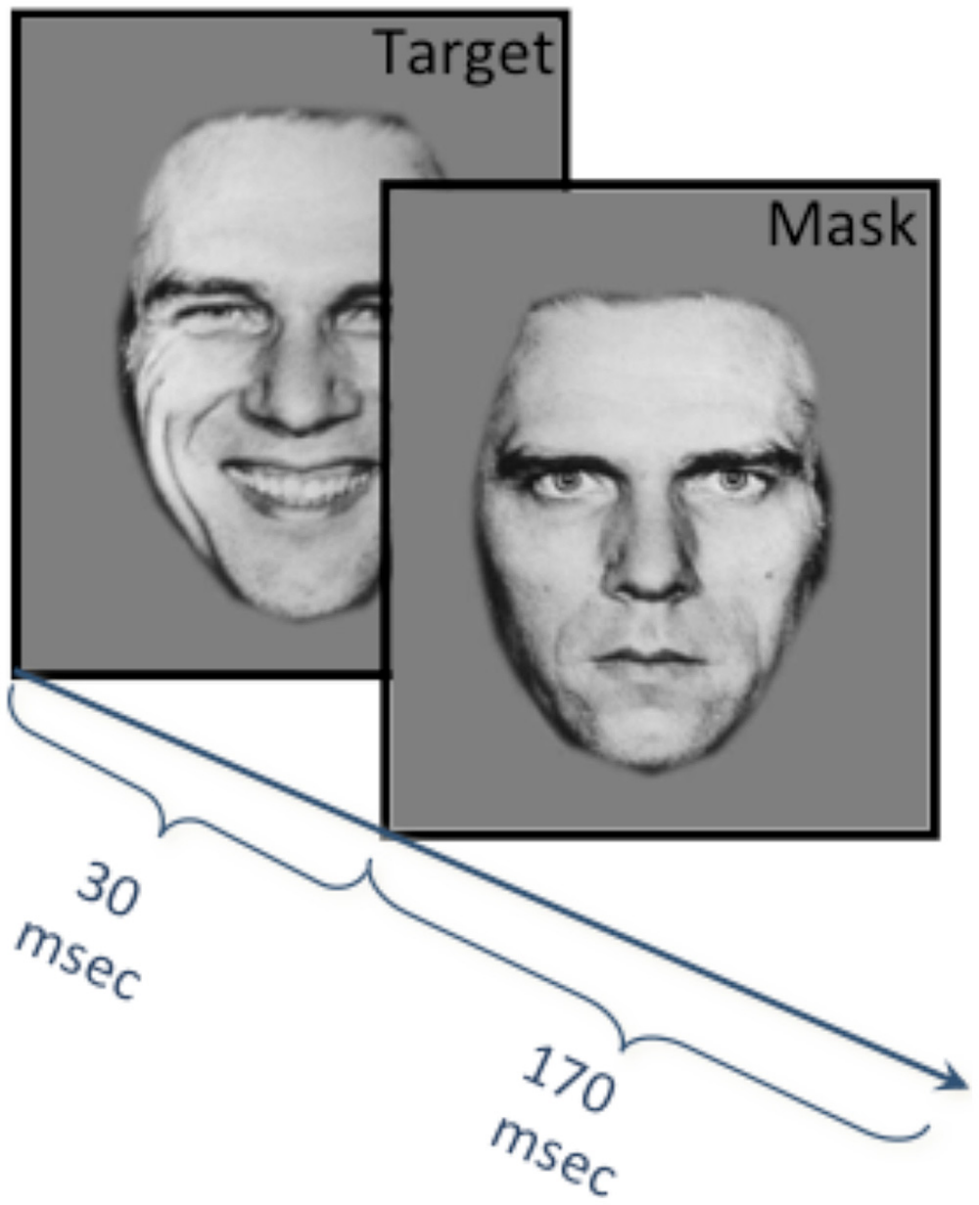
**Masked facial affect task methods.** Five alternating blocks of neutral (N) and emotional (E) masked target stimuli were presented during fMRI acquisition in the following fixed order: N,E,N,E,N. Each block consisted of 10 stimuli, and each stimulus consisted of either an emotion (happy angry or fearful) or neutral target face presented for 30 ms, followed immediately by a neutral masking face (i.e., neutral and emotion faced merged) for 170 ms with an inter-trial interval of 2800 ms. Emotional targets were matched for emotional state across all E blocks, and therefore each participant completed three runs of the task for each non-neutral emotional state (happy, fear, and anger).

As previously reported (Gruber et al., 2009; Sagar et al., 2013) the masked affect task was comprised of five alternating blocks of neutral (N) emotional (E) masked target stimuli in the following fixed order: N,E,N,E,N (**Figure 1**). Emotional targets were matched for emotional state across all E blocks of the task with no commingling of emotional stimuli type within a scanning epoch and only one emotional target type presented per scan. Therefore each participant completed three different runs of the masked facial affect task for each non-neutral emotional state (happy, anger, fear). Stimuli were presented this way to facilitate contrast analyses between the E and N blocks. Additionally, while individuals with BPD tend to view neutral faces as negative (Dyck et al., 2009; Ruocco et al., 2013), having the neutral mask across trials and using it as the single mask provides a consistent comparison across emotion target types.

Each of the five blocks consisted of ten trials. Each individual trial consisted of an emotional or neutral target face presented for 30 ms (“target”), followed immediately by a matched neutral masking face of the same person for 170 ms for a total combined stimuli presentation time of 200 ms. The intertrial interval was 2800 ms. Accordingly, each block of ten trials was 30 s long and an additional 6 s at the beginning of each scan was required for scanner calibration (no data were acquired during that time). Total scan time for each run of the task (happy, anger, fear) lasted 2 min and 36 s.

Participants were unaware of the backward masking nature of the paradigm, which attempts to limit the role of consciousness and appraisal processes. In order to ensure that they remained engaged, focused and attentive to the task, participants were told that they would see a series of briefly presented photographs of faces and were asked to make a gender discrimination for each face by pressing a small hand held key pad. Immediately upon completion of the functional scan, participants were presented with a post-test that included all facial expression stimuli and were asked to indicate for each expression whether it had been seen during the study. Participants were also asked to describe what they had seen of the presented faces and all reported the faces had neutral expressions, suggesting that the emotional target stimuli were presented below the level of conscious perception, and that individuals with BPD identified the faces as neutral. In addition, subjects in both groups were able to accurately report the gender of the neutral faces seen well above the level of chance, providing further evidence that they were actively engaged in the task.

### Image Acquisition and Analyses

Scanning was performed on a Siemens whole body 3T system using a quadrature head coil; 40 contiguous coronal slices were acquired from each subject to ensure whole brain coverage. Slices were 5 mm thick, with a 0 mm skip, and images were collected every 3 s (*TR* = 3000) using a single shot, gradient pulse echo sequence (*TE* = 30 ms, flip angle = 90, 50 images per slice). The task was presented using Psyscope 1.2.5 software generated from a Macintosh G5 computer and was rear projected onto a screen placed behind the top of the bore, visible through the mirror on the head coil. FMRI images were analyzed using a widely available software package SPM8 (Statistical Parametric Mapping, n.d.) running in Matlab (Matlab and Statistics Toolbox Release 2012b, 2012). Initially, blood oxygen level dependent (BOLD) fMRI data were corrected for motion in SPM8 using a 2-step intra-run realignment algorithm that uses the mean image created after the first realignment as a reference. A criterion of 3 mm of head motion in any direction was used as an exclusionary criterion; while no subjects had head movement that exceeded 3 mm, two subjects were removed from analyses due to poor image quality. The realigned images were then normalized to an EPI template in Montreal Neurological Institute (MNI) stereotactic space. Normalized images were re- sampled into 2 mm cubic voxels and then spatially smoothed using an isotropic Gaussian kernel with 8 mm full width at half maximum (FWHM). Global scaling was not used, high-pass temporal filtering with a cut-off of 128 s was applied, and serial autocorrelations were modeled with an AR(1) model in SPM8. Individual movement parameters were entered as regressors into the design. All regressors were convolved with a canonical hemodynamic response function.

Statistical parametric images were calculated individually for each participant and each task, using a general linear model (Friston et al., 1995). Individual first level contrast images were generated for the affect versus baseline contrast (FWE corrected, of 0.05). These images were subsequently entered into second level model, subjected to a voxel-wise contrast and *t*-test to assess statistical significance. Using a two-sample *t*-test, we made direct comparisons between the individuals with and without BPD. Double contrast analyses were conducted for each region of interest and for each task condition, which consisted of the subtraction of one group map from the other; for example, cingulate cortex activity of individuals with BPD during the viewing of angry faces was subtracted from cingulate cortex activity of the individuals without BPD viewing angry faces to determine which areas within the ROI showed increased activity in controls relative to those with BPD. Given the preliminary nature of this study, the statistical threshold was set at 0.05 uncorrected and a minimum cluster extent (k) of 10 contiguous voxels. These parameters were selected to maximize power and are consistent with the present study’s ROI hypothesis-driven approach. ROI masks were created using the Wake Forest University Pickatlas utility (Maldjian et al., 2003, 2004) and selecting appropriate anatomical regions. The ROI masks were defined as frontal, which included the selection of superior frontal, mid frontal and inferior frontal regions from the WFU Pickatlas utility; cingulate, which included the selection of mid and anterior cingulate, and the amygdala, which was dilated by an expanding factor of 1 to fully encompass the entire amygdala (Gianaros et al., 2008). Each of these regions has been identified as important for detecting, processing and evaluating emotionally relevant information, regardless of valence, in both research on masked affect processing and BPD. As previously reported (Gruber et al., 2009; Sagar et al., 2013) magnitude of neural activation was determined by the number of voxels activated within a specific ROI. Increased or “heightened” activation refers to a relatively higher number of voxels activated within an ROI for one group during a specific contrast relative to the other group.

## Results

Within-group data analyses indicated that across affective type (happy, anger, fear) versus neutral faces, individuals with BPD displayed a unique pattern of activation within the ROIs and a different number of voxels activated within the predefined ROIs (see **Table 1**).^1^ Below, we present double contrast analyses that compare activation patterns in individuals with and without a BPD diagnosis on affective versus neutral faces in key regions (see **Table 2**).^2^ As this preliminary investigation used an initial statistical threshold of 0.05 uncorrected, we completed Monte Carlo simulations in the AlphaSim module in AFNI (Ward, 2000) based on our ROI masks to compute the minimum voxel cluster size required to correct for Type I error in our statistical analysis. A minimum extent size of 98 continuous voxels was required for a corrected p value of 0.05, and accordingly, we have highlighted the contrasts that survived this correction within **Tables 1** and **2**.

**Table 1:** Masked affect fMRI results: single group comparisons.

REGION OF INTEREST Condition Group	Region	Cluster size (voxels)	*x*	*y*	*z*	SPM {t}	*p*-value
**FRONTAL**							
Happy-Neutral							
Non-BPD	Right superior frontal gyrus, BA10	37	24	66	18	2.54	0.005
BPD	Left superior frontal gyrus, BA10	**142**	-16	66	12	3.36	0.002
Anger-Neutral							
Non-BPD	Left inferior frontal gyrus, BA46	13	-40	34	12	2.21	0.019^+^
BPD	Left superior frontal gyrus, BA6	**139**	-12	-8	72	4.33	<0.001
Fear-Neutral							
Non-BPD	Left superior frontal gyrus, BA9	**100**	-14	52	26	4.25	<0.001
BPD	Left superior frontal gyrus	**120**	-14	-10	78	3.92	<0.001
**CINGULATE CORTEX**							
Happy-Neutral							
Non-BPD	Left midcingulate cortex	14	-8	-36	38	2.49	0.011
BPD	Left midcingulate cortex	**257**	-6	-4	36	3.01	0.001
Anger-Neutral							
Non-BPD	–	–	–	–	–	–	NS
BPD	Left midcingulate cortex	**252**	10	8	42	3.85	0.001
Fear-Neutral							
Non-BPD	Right anterior cingulate, BA24	**100**	4	34	2	2.90	0.004
BPD	Right midcingulate cortex	**177**	2	-14	42	3.23	0.002
**AMYGDALA**							
Happy-Neutral							
Non-BPD	–	–	–	–	–	–	NS
BPD	Right amygdala	**113**	30	6	16	3.24	0.001
Anger-Neutral							
Non-BPD	Left amygdala	57	-18	-2	-14	2.89	0.005
BPD	Right paraphippocampal gyrus, BA34	**130**	16	-2	-18	3.07	0.003
Fear-Neutral							
Non-BPD	–	–	–	–	–	–	NS
BPD	Left amygdala	10	34	4	-26	2.65	0.008

*^+^Signifies a trend-level effect using a 0.01 statistical threshold, but significant if using a 0.05 threshold.*

*Local maxima for group comparisons with frontal cortex, cingulate cortex, and amygdala regions of interest (ROI).*

***Bolded** cluster values indicate the contrast survived Monte Carlo simulation (*p* < 0.05) = 98 voxels, corrected.*

**Table 2:** Masked affect fMRI results: contrast analyses.

REGION OF INTEREST Condition Contrast	Region	Cluster Size (voxels)	*x*	*y*	*z*	SPM {t}	*p*-value
**FRONTAL**							
Happy-Neutral							
Non-BPD > BPD	Left superior frontal gyrus, BA9	**127**	-28	34	34	3.37	0.002
BPD > Non-BPD	Left inferior frontal gyrus	**132**	-44	16	8	2.91	0.004
Anger-Neutral							
Non-BPD > BPD	Right superior frontal gyrus, BA9	23	14	52	22	2.60	0.009
BPD > Non-BPD	Left superior frontal gyrus, BA6	**261**	-26	-2	70	3.81	0.001
Fear-Neutral							
Non-BPD > BPD	Left superior frontal gyrus, BA10	**107**	-22	46	22	3.71	0.001
BPD > Non-BPD	Right middle frontal gyrus, BA8	**211**	34	28	46	3.04	0.003
**CINGULATE CORTEX**							
Happy-Neutral							
Non-BPD > BPD	Right midcingulate cortex	39	12	-30	34	3.34	0.002
BPD > Non-BPD	Left midcingulate cortex	**99**	0	-4	34	3.16	0.002
Anger-Neutral							
Non-BPD > BPD	–	–	–	–	–	–	NS
BPD > Non-BPD	Left midcingulate cortex	29	-12	-4	44	3.43	0.001
Fear-Neutral							
Non-BPD > BPD	Right anterior cingulate, BA24	**120**	4	34	-2	3.21	0.002
BPD > Non-BPD	Right midcingulate cortex	**186**	2	-12	44	3.32	0.002
**AMYGDALA**							
Happy-Neutral							
Non-BPD > BPD	–	–	–	–	–	–	NS
BPD > Non-BPD	Right amygdala	89	32	-6	-14	3.25	0.002
Anger-Neutral							
Non-BPD > BPD	Left temporal pole	**100**	-30	4	-22	2.90	0.004
BPD > Non-BPD	–	–	–	–	–	–	NS
Fear-Neutral							
Non-BPD > BPD	–	–	–	–	–		NS
BPD > Non-BPD	Right middle temporal gyrus, BA38	**103**	34	4	-30	3.56	0.001

*Local maxima for group comparisons with frontal cortex, cingulate cortex, and amygdala ROI.*

***Bolded** cluster values indicate the contrast survived Monte Carlo simulation (*p* < 0.05) = 98 voxels, corrected.*

### Frontal

#### Happy

**FIGURE 2:**
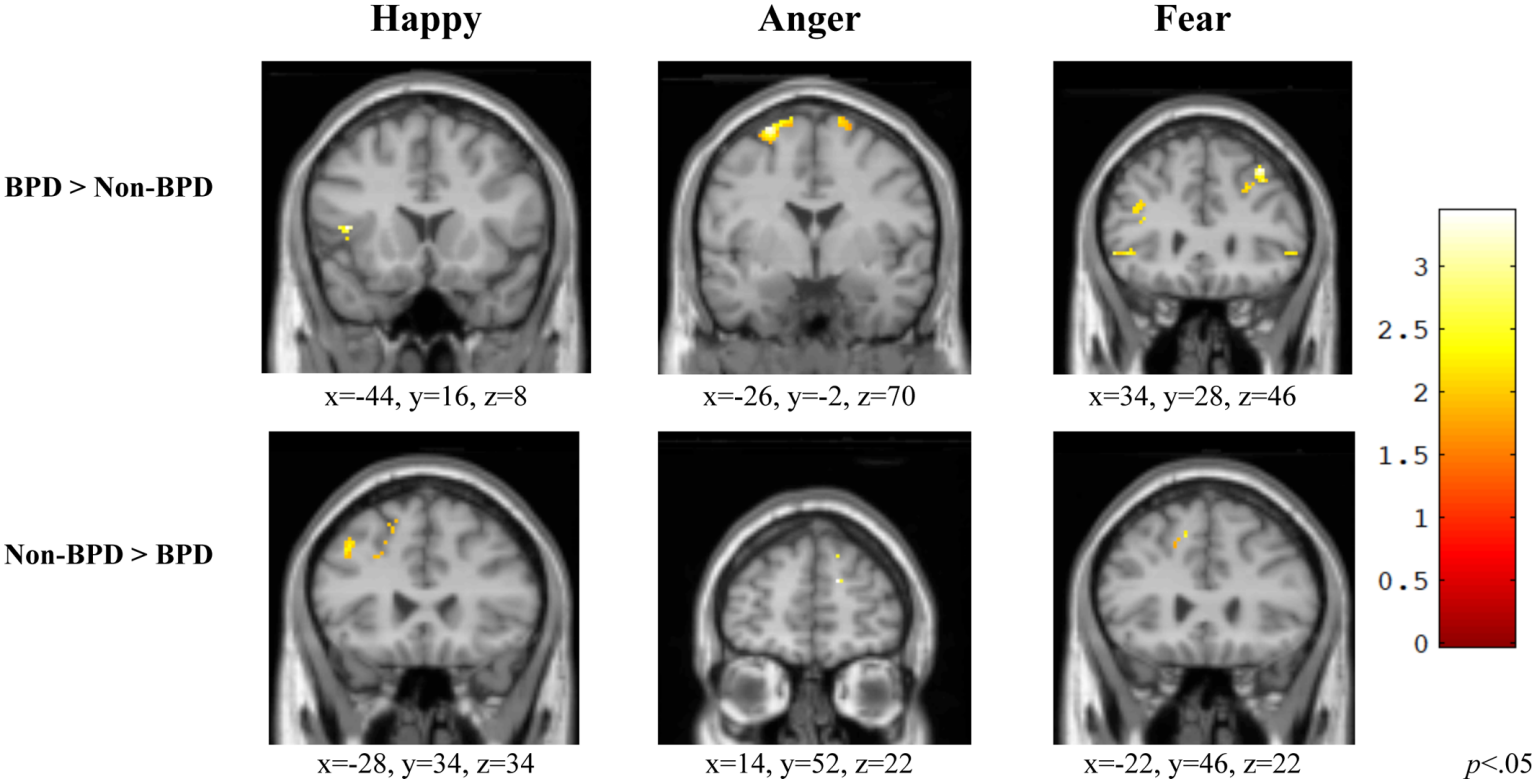
**Group comparisons of Frontal ROI activity in three affective categories.** Within the frontal ROI, individuals with BPD demonstrated a different pattern of activation relative to those without BPD and tended to display greater activation within the ROI relative to those without BPD. BPD, borderline personality disorder; Non-BPD, No diagnosis.

During the viewing of happy versus neutral faces, individuals diagnosed with BPD displayed significantly greater activation in the left inferior frontal gyrus [*t*(20) = 2.91, *p* = 0.004] relative to those without BPD. Individuals without BPD, however, displayed greater left superior frontal gyrus activation compared to those with the diagnosis [*t*(20) = 3.37, *p* = 0.002] (**Figure 2**, Happy).

#### Anger

When viewing angry versus neutral faces, individuals diagnosed with BPD displayed significantly greater left superior frontal gyrus activation [*t*(20) = 3.81, *p* = 0.001] than those without BPD. Individuals without BPD displayed greater right superior frontal gyrus activation compared to those with the diagnosis [*t*(20) = 2.60, *p* = 0.009] (**Figure 2**, Anger).

#### Fear

During the viewing of fearful versus neutral faces, individuals diagnosed with BPD displayed significantly greater activation in the right middle frontal gyrus [*t*(20) = 3.04, *p* = 0.003] as compared to those without BPD. However, individuals without BPD displayed greater left superior frontal gyrus activation compared to those with the diagnosis [*t*(20) = 3.71, *p* = 0.001] (**Figure 2**, Fear).

### Cingulate Cortex (CC)

#### Happy

**FIGURE 3:**
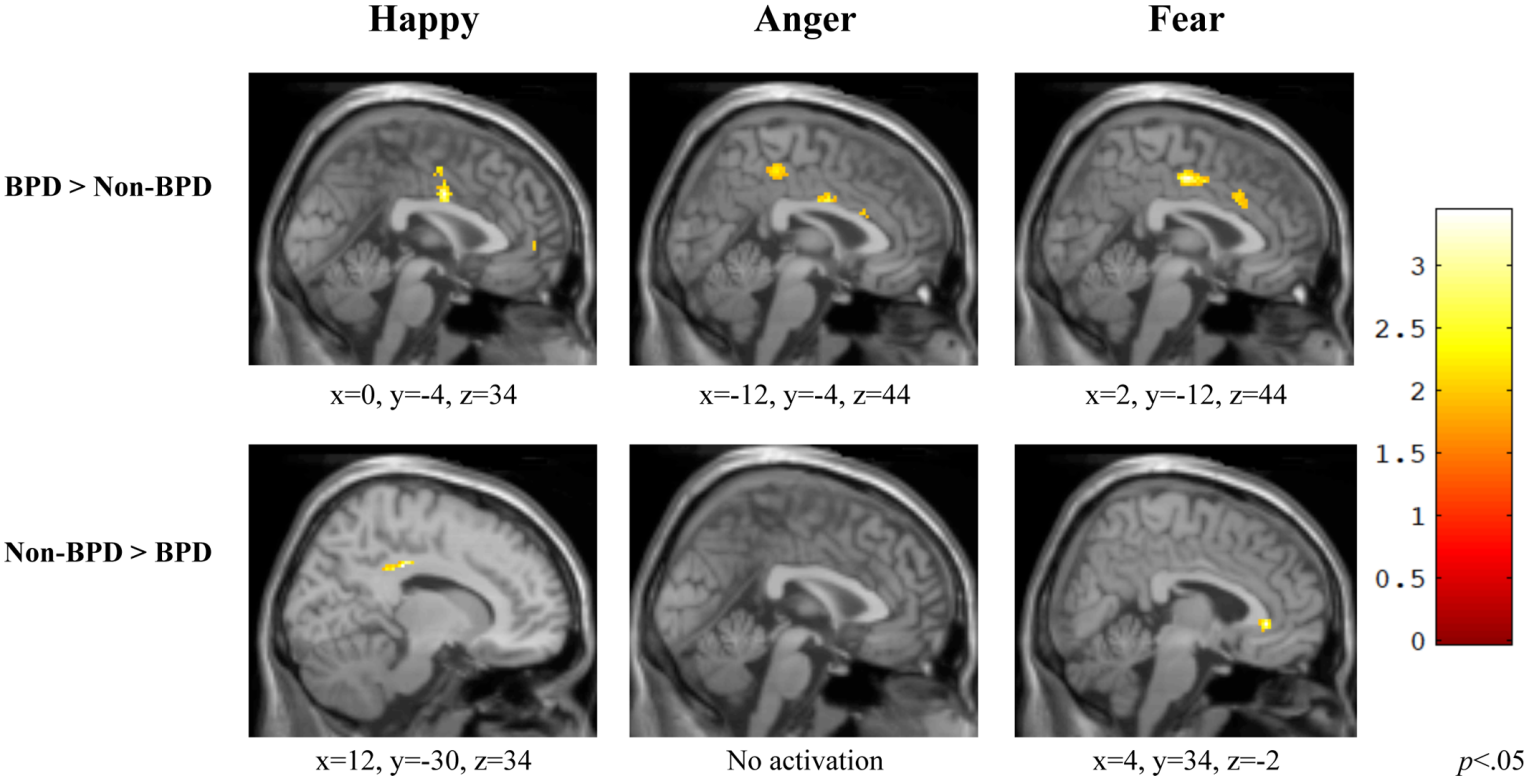
**Group comparisons of Cingulate Cortex ROI activity in three affective categories.** Across affective types, individuals diagnosed with BPD demonstrated a different pattern of activation than those without BPD, and exhibited greater midline cingulate activity within the ROI than those with no diagnosis. BPD, borderline personality disorder; Non-BPD, No diagnosis.

When exposed to happy versus neutral faces, individuals diagnosed with BPD displayed significantly greater activation in the left midcingulate (i.e., interhemispheric) CC [*t*(20) = 3.16, *p* = 0.002] relative to those without BPD. However, individuals without BPD displayed greater right midcingulate cortex activation compared to those with the diagnosis [*t*(20) = 3.34, *p* = 0.002] (**Figure 3**, Happy).

#### Anger

During the viewing of angry versus neutral faces, individuals diagnosed with BPD displayed significantly greater midline CC activity [*t*(20) = 3.43, *p* = 0.001] relative to those without BPD. For this contrast, individuals without BPD did not show greater activation anywhere in the CC compared to those with the diagnosis (**Figure 3**, Anger).

#### Fear

When viewing fearful versus neutral faces, individuals diagnosed with BPD displayed significantly greater activation in right midcingulate cortex [*t*(20) = 3.32, *p* = 0.002] relative to those without BPD. Individuals without BPD displayed greater right anterior cingulate activation compared to those with the diagnosis [*t*(20) = 3.21, *p* = 0.002] (**Figure 3**, Fear).

### Amygdala

#### Happy

**FIGURE 4:**
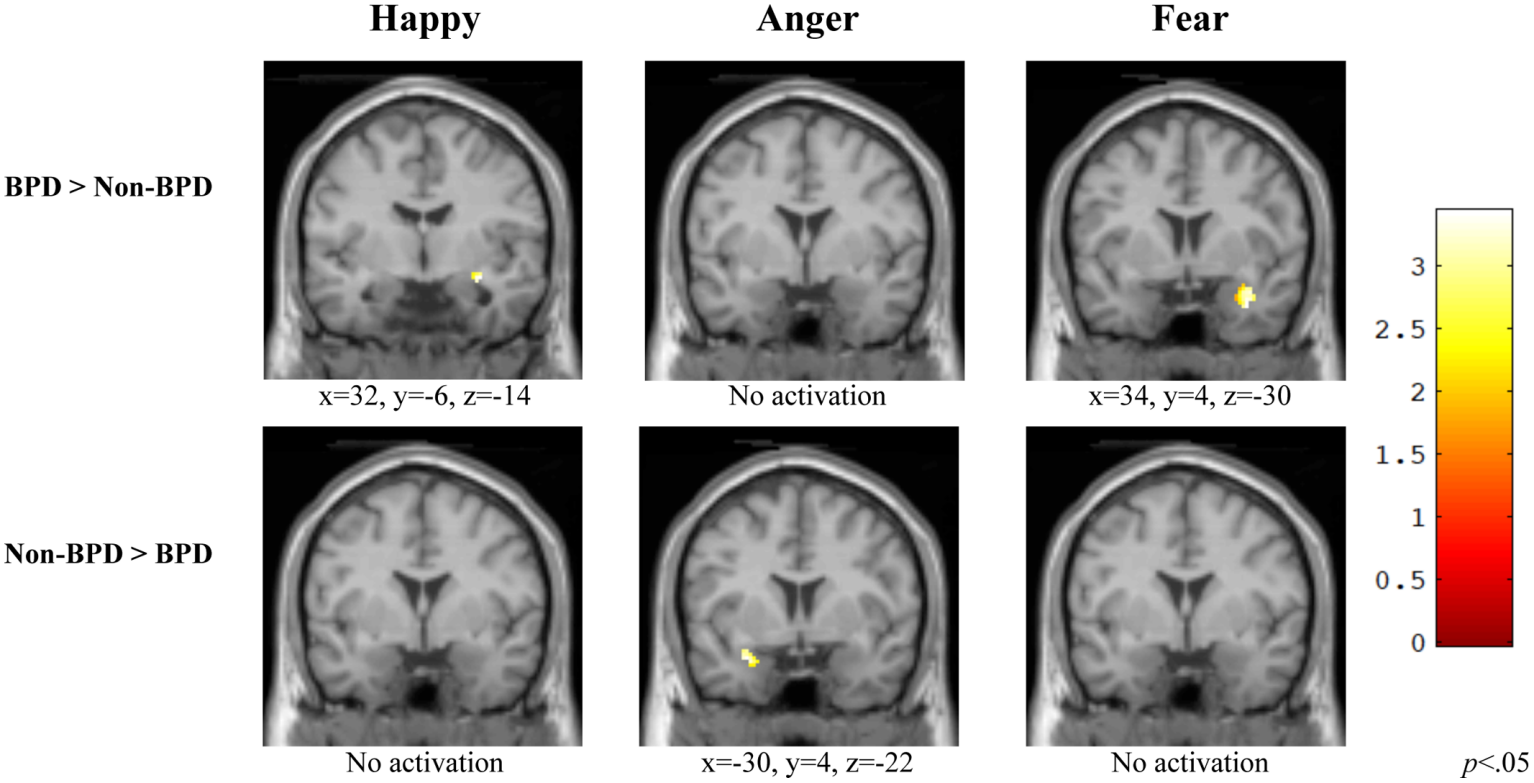
**Group comparisons of Amygdala ROI activity in three affective categories.** Within the amygdalar ROI, individuals diagnosed with BPD exhibited greater activation while viewing happy and fear versus neutral faces, but not while viewing anger versus neutral and had a differential pattern of activation overall relative to those without BPD. BPD, borderline personality disorder; Non-BPD, No diagnosis.

When viewing happy versus neutral faces, individuals diagnosed with BPD displayed significantly greater activation in the right amygdala [*t*(20) = 3.25, *p* = 0.002] relative to those without BPD. For this contrast, individuals without BPD did not show greater activation anywhere in the amygdala region compared to those with the diagnosis (**Figure 4**, Happy).

#### Anger

During the viewing of angry versus neutral faces, individuals with BPD did not show greater activation in the amygdala compared to those without the diagnosis. For this contrast, when compared to those without BPD, individuals with a BPD diagnosis displayed significantly greater activation in the left temporal pole [*t*(20) = 2.90, *p* = 0.004] (**Figure 4**, Anger).

#### Fear

During the viewing of fear versus neutral faces, individuals diagnosed with BPD displayed significantly greater activation in the right middle temporal gyrus [*t*(20) = 3.09, *p* = 0.002] relative to those without BPD. For this contrast, individuals without BPD did not show greater activation anywhere in the amygdalar region compared to those with the diagnosis (**Figure 4**, Fear).

## Discussion

Using a backward masked paradigm to investigate neural response to preattentively presented affective stimuli, we found that within selected ROIs, women with BPD displayed a different pattern of activity relative to healthy control women regardless of the affective condition, which was also accompanied by differences in the extent of activation (number of voxels) within these ROIs. Together, these findings indicate that individuals with BPD demonstrated heightened neural reactivity to affective information presented below the level of conscious awareness, and suggests the neural network involved in processing affect is distinct from those without BPD. To our knowledge, these data are the first to demonstrate differential neural activity to affective information presented below conscious awareness in BPD.

More specifically, findings within the frontal ROI were varied, both by group and by affective type. Consistent with findings that BPD patients engage a more diffuse network of neural structures associated with emotion processing and may have a deficiency in inhibitory control, individuals with BPD tended to show more posterior frontal activation, whereas those without BPD showed more anterior frontal activation. Within the CC, across affective expression, individuals with BPD exhibited a pattern of activation in the CC that was midline-mediated, suggesting increased attention to the affective stimuli. By contrast, individuals without BPD exhibited a more diverse pattern of anterior CC activation that was dependent on affective subtype, likely indicating discriminability of emotion regulatory functions. Finally, facial affect represented happy or fear expressions individuals diagnosed with BPD demonstrated greater activation within the amygdala ROI relative to those without BPD. However, consistent with evidence that individuals with BPD fail to differentiate anger from neutral expressions, individuals with BPD did not display greater amygdala activation to angry versus neutral faces than those without BPD.

Interestingly, the pattern of heightened neural response (i.e., greater activity within BPD group by region) to masked affective faces among females with BPD mirrors findings from studies using explicitly presented affective stimuli. Across methodologies (e.g., behavioral, imaging) and stimuli-type (e.g., word, image), individuals with BPD consistently demonstrate hyperreactivity to affective information (Rosenthal et al., 2008). However, while previous research indicates that the BPD-related emotion reactivity may be specific to negative valence (Arntz et al., 2000; c.f. Donegan et al., 2003; Koenigsberg et al., 2009; Hazlett et al., 2012), the present findings suggest the possibility their emotional reactivity may not be valence specific. Further research is needed to delineate the impact of valence on BPD-related emotional reactivity. Nevertheless, it is also possible that individuals with BPD are characterized by an underlying neurological vulnerability whereby they reflexively respond to and amplify all information perceived as affective, regardless of valence (Hooley and Gotlib, 2000; Daros et al., 2014). Their specific response to overt affect is likely modulated by existing biases, previous experiences, and cognitive functions, which may be stronger for negatively valenced information.

One advantage of a backward masked affect task is that it assesses perception of information prior to the impact of cognitive processing. Given this, the preattentive reactivity and altered neural responses associated with BPD may shape early stages of information processing. Results from the present study point to the possibility that those with BPD also implement a different neural network than those without BPD to process information. For example, contrast results indicated a midline mediated CC and posterior frontal activation in BPD versus more distributed CC and anterior frontal activation in non-BPD individuals. Consistent with previous work, this differential pattern of frontal region activation found in BPD individuals suggests that these individuals demonstrate widespread disruption in detecting, processing, and evaluating emotionally relevant information (see Rosenthal et al., 2008; Domes et al., 2009 for review). Additionally, within a specific location of the amygdala, individuals with BPD displayed significantly less differentiation between angry and neutral faces than those without BPD. Overall, single group analyses suggest that individuals with BPD displayed greater voxel-wise activation in the frontal CC, and amygdalar regions, but contrast analyses, designed to identify areas within the brain that individuals without BPD display more activation than those with BPD, indicated differences in neural activation and processing. The noted differences in both the magnitude and location of activation may reflect an inherent structural alteration that leaves individuals with BPD vulnerable to altered affective processing. This underlying processing vulnerability, though, is likely modulated by cognitive processes, such as selective attention and cognitive control (Breitmeyer and Ogmen, 2000). Therefore, once information reaches a level of consciousness, the interaction between their preattentive vulnerability and their cognitive biases may inform the behavioral response patterns associated with BPD. Such a proposal is consistent with prominent perspectives on BPD, which emphasize the importance of personal sensitivities for initiating dysregulated reactions.

According to Beauchaine et al. (2009) the affective dysregulation associated with BPD reflects a combination of developmentally acquired sensitivities combined with high trait impulsivity that confers a reduced capacity for cognitive control over such reactions. Alternatively, Selby and Joiner (2009) attribute the dysregulated emotion responses of individuals with BPD to an emotion cascade that involves intense rumination and negative affect in response to emotion-eliciting events (Selby and Joiner, 2009). In different ways, both of these theories suggest that the emotion dysregulation associated with BPD is not necessarily a function of the magnitude of the emotion response, but of the tendency for emotional stimuli to capture and hold the individual’s attention. Whereas the interpretation of affective stimuli and cognitive processes may be crucial in determining whether or not individuals with BPD display affective hyperreactivity, the impact of these stimuli on BPD may actually be a function of a more general sensitivity to perceiving emotion. That is, the heightened emotional reactions demonstrated by patients with BPD may occur because affective stimuli are more readily perceived, linked to pre-existing biases, and thus more likely to become interpreted as personally relevant and salient (Baskin-Sommers et al., 2012).

While findings from the present study are intriguing, several limitations should be considered. First, our sample size is small and findings from this study should be considered preliminary. As the current study is a preliminary investigation and we were interested in determining the patterns of activation during the processing of masked facial stimuli within each of the subject groups and the comparison of the groups to each other, regardless of the small sample size, the statistical threshold was set at 0.05 and a minimum cluster extent (k) of 10 contiguous voxels. While FWE corrections of 0.05 were made for the first level analyses, correction for multiple comparisons were not included in the current study given the sample size and specific hypotheses for the ROIs included in the manuscript. Bonferroni or similar corrections would likely be too conservative for a preliminary study of this size and could inflate Type II error rates, which could obscure potential signals arising from this small study with limited power. While this approach allowed us to identify differential processing patterns for the groups during the task, we completed Monte Carlo simulations based on our ROI masks in order to compute the minimum voxel cluster size required to correct for potential Type I errors in our statistical analysis. Results of the simulations indicated that a minimum extent size of 98 continuous voxels was required for a corrected p value of 0.05. As illustrated in **Table 2**, the majority of contrasts (8/12) met or exceeded this threshold. Nevertheless, results from the current preliminary investigation must be interpreted with caution, and additional research studies with larger sample sizes are needed to confirm these findings. Second, this study included only female subjects, and although this approach has the advantage of decreasing the heterogeneity of the sample, it is unknown whether males with BPD would exhibit a similar pattern in response to masked affective faces. Third, as is typical of people with this disorder, many BPD participants were taking antidepressant medications, which may impact neural functioning, particularly within limbic regions (see Victor et al., 2013 for a study in Major Depression). Additionally, most participants met criteria for major depressive disorder (but see Footnotes 1 and 2). Psychiatric comorbidity is common in BPD (Zanarini et al., 1998); however, future research should examine the role that medications and comorbid diagnoses may play in preattentive emotion processing. Further, findings from the current study do not allow us to examine activation in additional regions involved in emotion processing (e.g., insula; see Ruocco et al., 2013) or early visual processing areas. However, previous work has suggested that the insula may be important for understanding the range of affective dysfunctions related to BPD. Additionally, research has suggested that the amygdala has greater responsivity to intact or low spatial frequency stimuli relative to high spatial frequency facial stimuli in healthy control subjects (Vuilleumier et al., 2003). As this has not yet been explored in individuals with BPD, it remains unknown whether those with BPD demonstrate a perceptual sensitivity, manifested by increased responsivity to low level visual information resulting in increased activation within the amygdala. Future studies with larger sample sizes should examine additional neural regions relevant to affective processing, as well as, high versus low spatial facial stimuli in BPD. Lastly, this study did not include a measure of overt affect discrimination (e.g., Facial Affect Discrimination task), therefore, it is impossible to evaluate whether the present BPD-related preattentive neural hypersensitivity is related to overt affective hypersensitivity, recognition, or responsivity in this sample. Future studies should examine associations between preconscious and conscious processing.

Despite these limitations, data from this preliminary study extends our current understanding of the process- and neural-level correlates of BPD. In BPD, heightened activation to preattentive affective faces may predispose these individuals to hypervigilance, increase orienting to salient features of these stimuli, and reduce opportunities to regulate a reaction to these important social cues. Additionally, the perception or misperception of subtle fluctuations in the emotional expressions of others, both positive and negative, could substantially contribute to the emotional volatility of individuals with BPD. From a clinical perspective, this reflexive responsivity to emotion underscores the importance of helping individuals with BPD overcome their vulnerability by developing a more balanced appraisal of other people’s emotions, encouraging them to tolerate their own initial reactivity without needing to act on it, and moving toward getting these individuals to accept their tendency to perceive information in an affectively laden manner.

## Authour Contributions

Conceptualization: JH, DY-T, SG; Methodology: DY-T, SG, JH, MD, AG; Investigation: SG, MD; Formal Analysis: SG, AG; Writing-Original Draft: AB-S; Writing-Reviewing and Editing: DY-T, SG, JH, AB-S; Supervision: SG; Project Administration: SG; Resources: JH.

## Conflict of Interest Statement

The authors declare that the research was conducted in the absence of any commercial or financial relationships that could be construed as a potential conflict of interest.

## Footnotes

1 As noted in the Methods, there was a high rate of comorbidity between BPD and depression in the present sample. Even though this is commonly observed in BPD samples (Zanarini et al., 1998), it is possible that neural differences between BPD and Non-BPD individuals are due to depressive symptomatology, rather than BPD. In order to evaluate this possibility, we reanalyzed the within group contrasts with Beck Depression Inventory (BDI) total score as a continuous covariate (Mean_BPD_ = 21 Mean_Non-BPD_ = 1, between-group *p* < 0.001). The results remained largely the same. Slight differences were observed in the Non-BPD diagnosed group, where the Anger-Neutral Frontal and Happy-Neutral CC effect were no longer significant, after controlling for BDI score. One difference was seen in the BPD group, where the Anger-Neutral Amygdala contrast was no longer significant, after controlling for BDI score. Of note, this latter non-significant effect is consistent with the reported double contrast, where there was no area that the BPD group activated more than Non-BPD group in the amygdala for Anger versus Neutral trials.

2 We reanalyzed the double contrast analyses with BDI score as a continuous covariate. All of the effects where the BPD group activated more voxels than the Non-BPD group remained identical. Three differences were observed for the Non-BPD greater than BPD results. First, the Anger-Neutral Frontal effect was non-significant after controlling for BDI score, suggesting that the Non-BPD group no longer activated more voxels than the BPD group in this contrast and region. Second, the Happy-Neutral CC effect was non-signficant after controlling for BDI score. Finally, the previously non-significant effect of Anger-Neutral CC in the Non-BPD versus BPD group was now significant, after controlling for BDI score. Specifically, the Non-BPD group activated more voxels than the BPD group within the CC ROI specifically within the medial CC (*x* = 10, *y* = 16, *z* = 44, *k* = 76, *t* = 3.54, *p* = 0.001). Overall, there was little change in the results after controlling for BDI score.
